# Pregabalin acts on Na^+^–Ca^2+^ exchanger, which promotes Ca^2+^ extrusion from human Merkel cell line

**DOI:** 10.1097/PR9.0000000000001381

**Published:** 2025-12-18

**Authors:** Rumi Kaneko, Takehito Ouchi, Maki Kimura, Hidetaka Kuroda, Tatsuya Ichinohe, Yoshiyuki Shibukawa

**Affiliations:** a Department of Physiology, Tokyo Dental College, Chiyoda-ku, Tokyo, Japan; b Department of Dental Anesthesiology, Tokyo Dental College, Chiyoda-ku, Tokyo, Japan; c Department of Dental Anesthesiology, Kanagawa Dental University, Yokosuka, Kanagawa, Japan

**Keywords:** Neuropathic pain, Pregabalin, Na^+^–Ca^2+^ exchanger, Pharmacological agent

## Abstract

**Introduction:**

Pregabalin, a first-line treatment for neuropathic pain, binds to the voltage-gated calcium channel auxiliary subunit alpha2delta-1, reducing neurotransmitter release. Transdermal pregabalin has also shown analgesic effects in neuropathic mouse models. However, its effects on epidermal cells remain unclear.

**Objectives:**

We aimed to investigate the action of pregabalin on the human Merkel cell line (MC).

**Methods:**

We used the cultured human Merkel cell line MCC 14/2 to measure intracellular free calcium concentration ([Ca^2+^]_i_) levels using fura-2 and to perform immunofluorescence analysis.

**Results:**

To examine effect of pregabalin on a chronic pain condition, we applied Lys-(Des-Arg^9^) bradykinin (BK) for 48 hours (BK group), BK for 48 hours with pregabalin for 24 hours (BK + pregabalin group), or none (as control group) before measuring the direct mechanical stimulation–induced [Ca^2+^]_i_ response in the MCs. The area under the curve (AUC) of the transient increase in [Ca^2+^]_i_ was significantly increased in the BK group than that in the control group. Area under the curve did not show any significant differences between control group and BK + pregabalin group. When the Na^+^–Ca^2+^ exchanger (NCX) inhibitor KB-R7943 and SEA0400 were applied just before mechanical stimulation in the BK + pregabalin group, AUC was significantly increased compared to that in the absence of KB-R7943 and SEA0400. We could not observe any significant differences in the peak values of mechanical stimulation–induced [Ca^2+^]_i_ increases among the groups. Merkel cells predominantly expressed the NCX1 isoform.

**Conclusion:**

These results suggested that the site of action of pregabalin to MCs is Ca^2+^ extrusion mechanism via NCX1.

## Abstract 2

Supplemental Digital Content is Available in the Text.

Pregabalin modulates intracellular Ca^2+^ homeostasis by increasing Ca^2+^ extrusion activity via the plasma membrane Na^+^–Ca^2+^ exchanger in Merkel cells.

## 1. Introduction

Neuropathic pain is defined as “pain caused by a lesion or disease of the somatosensory system”^18^ and includes not only spontaneous pain but also hyperalgesia (a state of heightened response to painful stimuli) and allodynia (symptoms of pain induced by a stimulus which normally does not cause pain).^19^ Pregabalin is recommended as a first-line pharmacological agent for managing neuropathic pain.^29^ Pregabalin acts on the voltage-gated Ca^2+^ channel auxiliary subunit alpha2delta-1 (Cacna2d1) in the presynaptic terminals of neurons in the brain and spinal cord. It reduces the synaptic release of neurotransmitters by binding to Cacna2d1.^20^ However, patients treated with internal use of pregabalin may experience severe central nervous system–mediated side effects, such as dizziness and somnolence. Topical application of pregabalin via the transdermal route is expected to reduce systemic drug exposure and side effects; the effectiveness of its transdermal administration has been reported recently. An aqueous solution of pregabalin was shown to have an analgesic effect 30 minutes to 1 hour after transdermal application to the hind feet in a rat model of partial sciatic nerve ligation and mouse model of spinal nerve ligation.^12^ When a pluronic lecithin organogel formulation of pregabalin was applied to the feet skin of a mouse model of diabetic neuropathy, pregabalin was found to be distributed near the dermis 1 hour after application, and an analgesic effect was observed.^30^ In addition, for patients suffering from orofacial pain after tooth extraction or endodontic treatment, a topical preparation containing 10% pregabalin was applied intraorally and held in place using a stent or drug delivery device.^14^ Topical medications have an analgesic effect for 5 minutes after application in patients suffering from neuropathic pain and allodynia or hyperalgesia.^14^ Allodynia, also known as touch-induced pain, might be closely associated with the stimulation of epithelial touch receptors. Therefore, pregabalin might act not only on the presynaptic terminals of neurons but also on epithelial touch receptors in peripheral tissues.

Merkel cells (MCs) are sensory receptors in the touch-sensitive areas of the glabrous epithelium, such as the outer root sheaths of hair follicles and the oral mucosa.^4^ When mechanical stimulation is applied to sensory receptors, intracellular Ca^2+^ concentration ([Ca^2+^]_i_) increases via various mechanosensitive ion channels, such as piezo channels. After Ca^2+^ influx via mechanosensitive ion channels, [Ca^2+^]_i_ levels are maintained by the Ca^2+^ extrusion system, including the Na^+^–Ca^2+^ exchanger (NCX) and plasma membrane Ca^2+^-ATPase (PMCA).^5^ [Ca^2+^]_i_ is very precisely regulated, since [Ca^2+^]_i_ overload results in cytotoxicity.^7^ Such an overload of [Ca^2+^]_i_ due to decreased expression of K^+^-dependent Na^+^–Ca^2+^ exchanger, eg, is reported to be associated with neuropathic pain.^40^

The present study aimed to clarify the cellular mechanism of transdermal application of pregabalin to relieve neuropathic/nociceptive pain. For this purpose, we focused on the MCs and analyzed Ca^2+^ extrusion kinetics after direct mechanical stimulation–induced [Ca^2+^]_i_ increase.

## 2. Methods

### 2.1. Cell culture

Merkel cell carcinoma cell line MCC 14/2 was established from a skin lesion on the foot of an 80-year-old man and was reported to show the general properties of MCs.^3,21,22,24,33,36^ MCC 14/2, designated as MCs in this study, was cultured in RPMI 1640 medium (ATCC modification) (Thermo Fisher Scientific, Waltham, MA) supplemented with 10% fetal bovine serum, 100 U/mL penicillin–streptomycin (Thermo Fisher Scientific), and amphotericin B (Sigma-Aldrich, St. Louis, MO) at 37°C in a 5% CO_2_ incubator for 48 hours. The MC suspension was adjusted to a density of 2 × 10^4^ cells/mL in 35 mm dishes (ibidi GmbH, Gräfelfing, Germany).

### 2.2. Solutions and reagents

Standard extracellular solution (ECS) contained (in mM) 136 NaCl, 5 KCl, 2.5 CaCl_2_, 0.5 MgCl_2_, 10 HEPES, 10 glucose, and 12 NaHCO_3_ (pH 7.4). High-K^+^ solution had 50 mM of KCl with reduced NaCl (91 mM). Full details are in Supplementary Methods, http://links.lww.com/PR9/A366.

### 2.3. Immunofluorescence

Cells were fixed with paraformaldehyde, permeabilized, and blocked at room temperature. Primary antibodies were incubated overnight at 4°C. Full details are in Supplementary Methods, http://links.lww.com/PR9/A366.

### 2.4. Measurement of intracellular free Ca^2+^ concentration

Fura-2 fluorescence was measured at 510 nm with alternating excitation at 340 nm (F340) and 380 nm (F380). [Ca^2+^]_i_ was assessed using the fluorescence ratio value (R_F340/F380_ value; F), normalized (*F/F*_*0*_) to baseline value (*F*_0_). Full details are in the Supplementary Methods, http://links.lww.com/PR9/A366.

### 2.5. Direct mechanical stimulation of single Merkel cell

Direct mechanical stimulation was applied to single MCs using a fire-polished glass micropipette (GC150F-10; Harvard Apparatus, Holliston, MA, tip diameter 2–3 μm), which was pulled from the capillary tubes using a DMZ universal puller (Zeitz Instruments, Martinsried, Germany) and filled with standard ECS. The micropipette was operated using a micromanipulator (UMp Micromanipulators; Sensapex, Oulu, Finland). The tip was positioned just above the target cell membrane, after which a focal mechanical stimulation was performed by moving the micropipette 6.0 μm vertically downward at a speed of 2.0 μm/second. Stimulation was applied for 6 seconds, and the pipette was moved back to its original position at the same velocity.

### 2.6. Statistical analysis

For [Ca^2+^]_i_ measurements, *F/F*_*0*_ unit, as [Ca^2+^]_i_, was obtained as an average from 10 to 30 cells per experiment during depolarizing stimulation, whereas it was measured from a single cell per experiment during mechanical stimulation. Data are expressed as mean ± standard errors (SE) or standard deviation (SD) of the mean of N observations, where N represents the number of experiments or cells tested. To analyze changes in [Ca^2+^]_i_, the Shapiro–Wilk test was used to assess for normality. Nonparametric statistical significance was determined using the Mann–Whitney test, the Kruskal–Wallis test, or the Friedman test, followed by Dunn multiple comparison test. Parametric statistical significance was determined using Welch *t* test and ordinary 1-way ANOVA followed by Tukey and Dunnett multiple comparison test. Statistical analyses are detailed in the Figure Legends. Statistical significance was set at *P* < 0.05. Results of independent 1-way ANOVA were reported along with F-values and corresponding degrees of freedom. All statistical analyses were performed using GraphPad Prism 8.0 (GraphPad Software, La Jolla, CA). Graphical representations (ie, graphs and traces) were prepared using data analyzed in Origin 8.5 (OriginLab Corporation, Northampton, MA).

## 3. Results

### 3.1. Immunofluorescence analysis of Merkel cells

**Figure 1.**
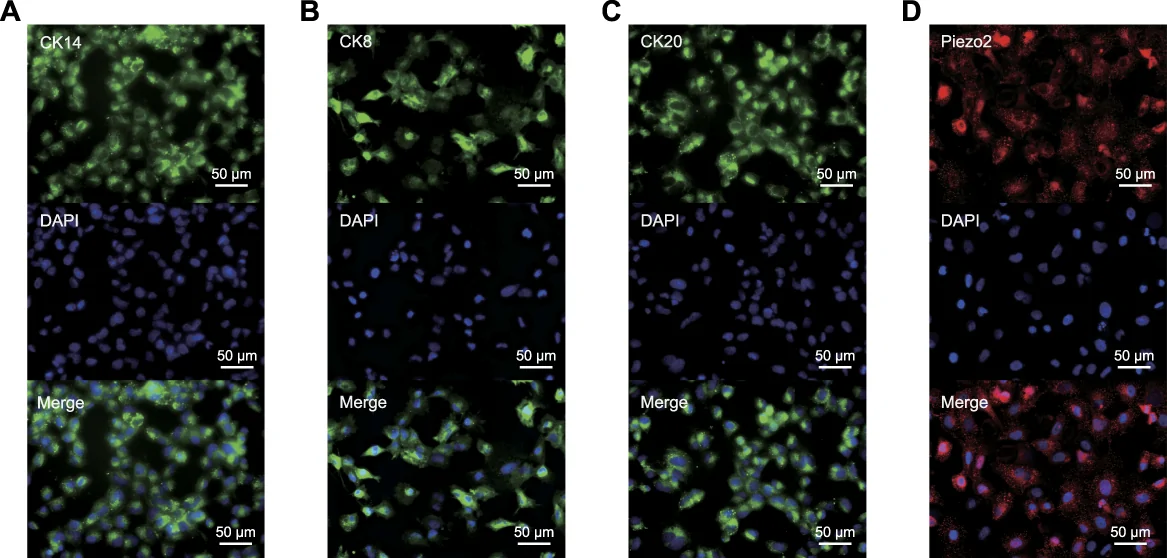
Expression of cytokeratin (CK) 14, 8, 20, and Piezo2 channel in human Merkel cells (MCs). (A–D) Human MCs exhibited positive immunoreactivity to CK 14 (green; upper panel in A), CK 8 (green; upper panel in B), CK 20 (green; upper panel in C), and Piezo2 (red; upper panel in D) antibodies. 4,6-diamidino-2-phenylindole (DAPI) was used to stain the nuclei. (blue: middle panels in A to D). The lower panels in (A-D) display the merged images. Scale bars: 50 μm. No fluorescence was detected in the negative controls (not shown).

To evaluate the protein expression patterns of cultured MCC 14/2 cells, we performed immunofluorescence analysis using the epithelial cell marker cytokeratin (CK) 14.^2^ Further, we evaluated immunofluorescence of MC marker proteins CK8 and 20.^28^ The cultured cells were immunopositive for all markers localized on cell membrane and/or on cytoplasm (green in Figs. 1A–C), suggesting that they have the characteristics of MCs. In addition, we observed immunoreactivity for the Piezo2 channel on the MCC 14/2 cells (red in Fig. 1D). No fluorescence was detected in the negative control in which the primary antibody was omitted and buffer was used instead (not shown).

### 3.2. Direct mechanical stimulation of Merkel cells transiently increased [Ca^2+^]_i_

**Figure 2.**
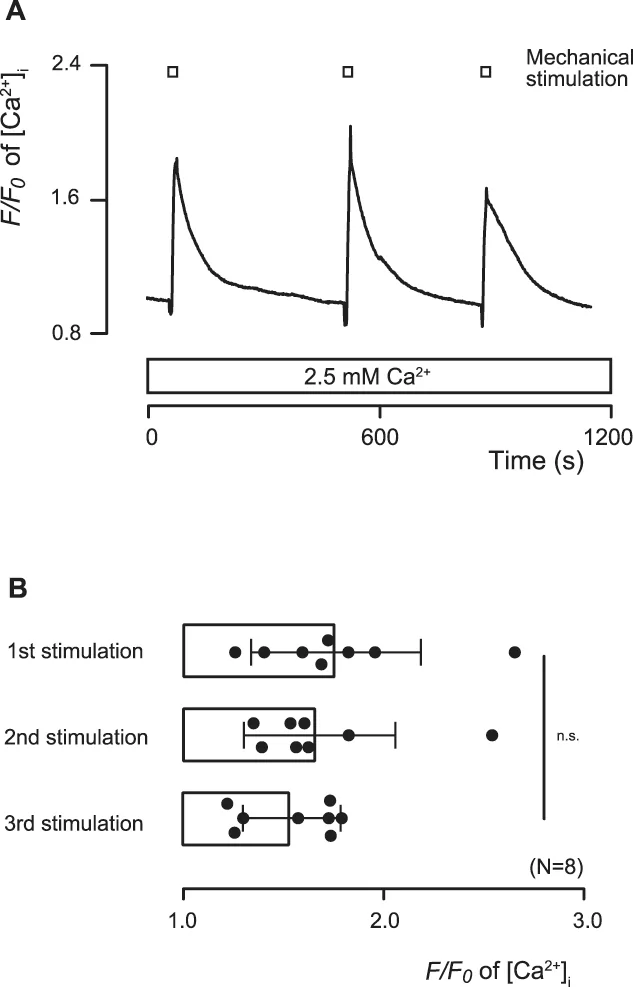
Transient increases in [Ca^2+^]_i_ by direct mechanical stimulation of human MCs. (A) Representative trace shows transient increases in [Ca^2+^]_i_ during repeated mechanical stimulations (upper white boxes) in the presence of extracellular Ca^2+^ (lower white box). (B) Bar graph shows the *F/F*_*0*_ values as a function of the number of series applications in the mechanical stimuli by vertical micropipette displacement (6.0 μm). Each bar denotes the mean ± SD of 8 experiments, and each black circle represents each *F/F*_*0*_ value. No statistically significant difference between columns (solid lines) is indicated with n.s.; data were analyzed by the Friedman test followed by Dunn multiple comparison test. MCs, Merkel cells.

After culturing MCs for 48 hours at 37°C in 5% CO_2_, we measured the fura-2 fluorescence as [Ca^2+^]_i_. When we applied mechanical stimulations to MCs using a glass micropipette by moving it vertically (6 μm) in a downward direction from a position just above the surface (0 μm) in the presence of extracellular Ca^2+^ (N = 8), we observed transient [Ca^2+^]_i_ increase (Fig. 2A). After [Ca^2+^]_i_ returned to near-resting levels, further mechanical stimulation increased [Ca^2+^]_i_ transiently in the MCs. Repeated increases in transient [Ca^2+^]_i_ were observed upon mechanical stimulation of MCs; however, no desensitizing effects on [Ca^2+^]_i_ increases were observed (*P* > 0.05; Figs. 2A, B). Data were analyzed by the Friedman test followed by Dunn multiple comparison test.

### 3.3. Pregabalin did not suppress [Ca^2+^]_i_ increase and Ca^2+^ extrusion kinetics of cells after direct mechanical stimulation to Merkel cells

**Figure 3.**
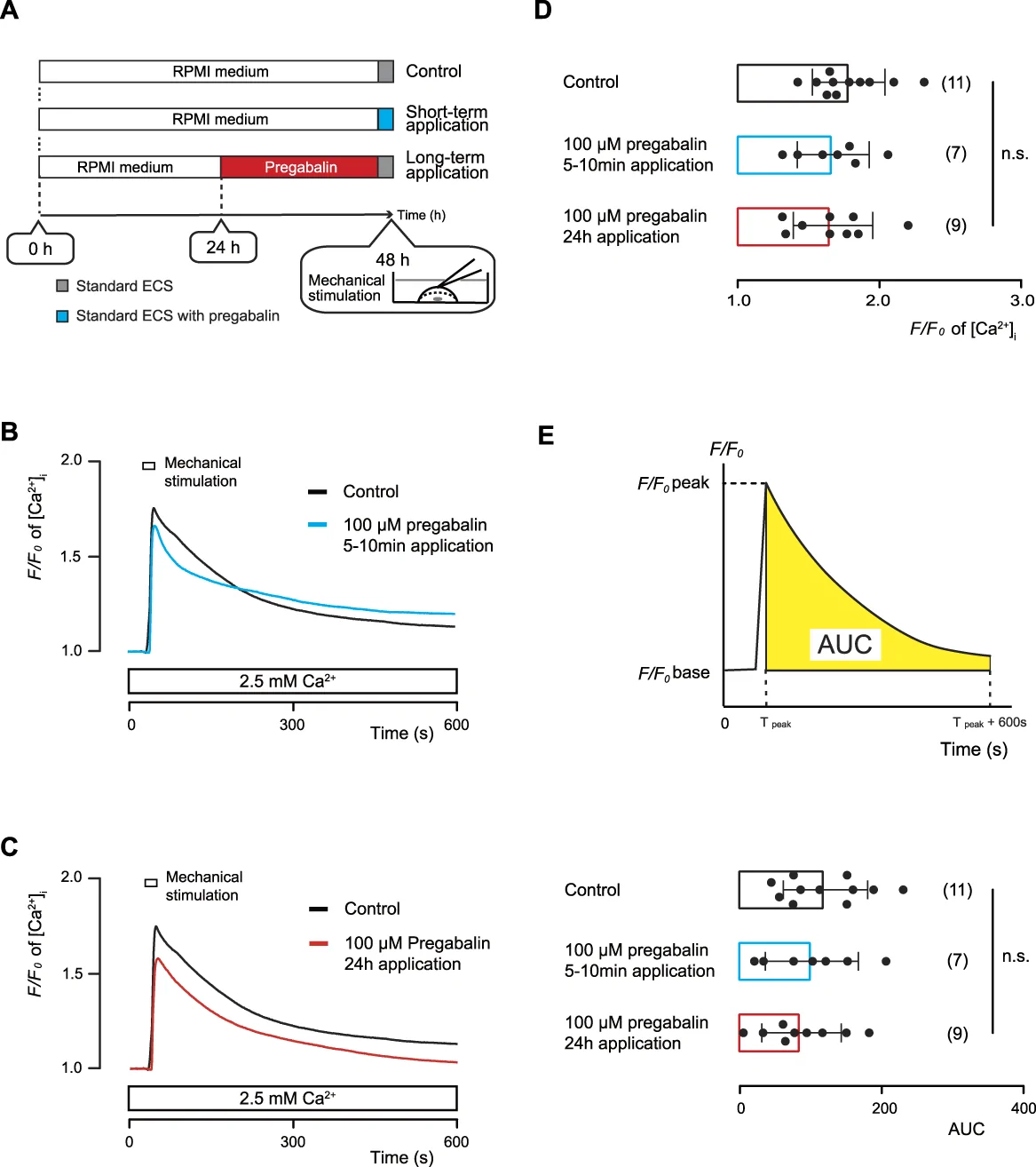
Effects of pregabalin on direct mechanical stimulation–induced Ca^2+^ influx in human MCs. (A) The protocol shows the application of a pharmacological agent to the MC culture. Each box indicates the time course of pharmacological reagents applied in this experiment (gray, standard ECS; blue, 5–10 minutes application of standard ECS with 100-μM pregabalin; red, culture condition with RPMI medium including 100-μM pregabalin). White boxes indicate culture condition with RPMI medium. Upper represents control condition; direct mechanical stimulations were applied to MCs that were cultured in RPMI medium for 48 hours. Middle shows the short-term application of pregabalin; MCs were mechanically stimulated after the application of 100-μM pregabalin for 5 to 10 minutes after 48-hour cultivation in RPMI medium. Lower represents the long-term application of pregabalin; MCs were cultured in RPMI medium for 24 hours followed by 24-hour culture in RPMI containing 100-μM pregabalin, before direct mechanical stimulation. (B–E) Direct mechanical stimulations were induced by vertical downward displacement of micropipette by 6.0 μm (white boxes at the top in B and C) under control conditions (N = 11, black line in B and C, and black columns in D and E [lower graph]). Pregabalin (100 μM) was applied for 5 to 10 minutes as short-term application (N = 7, blue line in B and blue columns in D and E [lower graph]) or for 24 hours as long-term application (N = 9, red line in C and red columns in D and E [lower graph]) to MCs. Traces (B and C) obtained by averaging the independent traces (black line: N = 11, blue line: N = 7, red line: N = 9) of transient increase in [Ca^2+^]_i_ during mechanical stimulation for each condition. Note that the black lines in (B and C) are identical, making it easier to understand. Bar graphs in (D) show the peak values of [Ca^2+^]_i_ increase induced by direct mechanical stimulation (up to 6.0 μm). To evaluate the Ca^2+^ extrusion efficacies after the peak [Ca^2+^]_i_ increase, we analyzed the area under the curve (AUC, inset of E) of the transient increase in [Ca^2+^]_i_ during 600-second time period from the time showing peak *F/F*_*0*_ value (T_peak_) to time of (T_peak_ + 600 seconds). Bar graph in (E) shows AUC in each condition. Each bar in (D and E) denotes the mean ± SD across the number of experiments shown in parenthesis, and each black circle represents each *F/F*_*0*_ value or AUC. Statistically significant differences between columns (solid lines) are indicated by asterisks; *P < 0.05; n.s., not significant. Statistical analyses were performed using the ordinary 1-way ANOVA followed by Dunnett multiple comparison test in (D and E). ECS, extracellular solution; MCs, Merkel cells.

Pregabalin was applied in 2 ways as describing below (Fig. 3A). One was short-term application; after 48 hours culturing MCs with RPMI medium, 100-μM pregabalin was applied for 5 to 10 minutes to MCs just before direct mechanical stimulation. Another was long-term application; during 48 hours of culturing in total, cells were cultured for 24 hours in RPMI medium with 100-μM pregabalin after a 24-hour culture without pregabalin, and we applied direct mechanical stimulation to the MCs.

In the presence of extracellular Ca^2+^ (2.5 mM), the application of direct mechanical stimulation to MCs (6.0-μm depth) resulted in a transient increase in [Ca^2+^]_i_ (black line in Figs. 3B, C) up to peak value of 1.72 ± 0.26 in *F/F*_*0*_ units (N = 11, black column in Fig. 3D) under control conditions. The peak value of [Ca^2+^]_i_ increase, induced by mechanical stimulation, was inhibited by neither the short-term application (F_(2,24)_ = 0.80, *P* = 0.62; N = 7, blue line in Fig. 3B and blue column in Fig. 3D) nor long-term application (F_(2,24)_ = 0.80, *P* = 0.56; N = 9, red line in Fig. 3C and red column in Fig. 3D) of 100-μM pregabalin. The peak values of mechanical stimulation–induced [Ca^2+^]_i_ increases were 1.55 ± 0.17 in *F/F*_*0*_ units for short-term application and 1.63 ± 0.21 in *F/F*_*0*_ units for long-term application. To evaluate the Ca^2+^ extrusion efficacies after the peak [Ca^2+^]_i_ increase, we measured and analyzed the area under the curve (AUC) of the transient increase in [Ca^2+^]_i_ during 600 seconds from the time showing peak value of *F/F*_*0*_ (T_peak_) (ie, the time period from T_peak_ to “Tpeak + 600 seconds”) (inset of Fig. 3E). There were no significant differences in the value of AUC among control (120.9 ± 60.0 [N = 11]; black column in Fig. 3E [lower graph] as control), short-term application (101.5 ± 65.9 [N = 7]; blue column in Fig. 3E, F_(2,24)_ = 0.57, *P* = 0.62), and long-term application (86.9 ± 56.2 [N = 9]; red column in Fig. 3E, F_(2,24)_ = 0.57, *P* = 0.56). Statistical analyses were performed using the ordinary one-way ANOVA followed by Dunnett multiple comparison test for the peak values and the value of AUC.

### 3.4. Pregabalin decreased area under the curve after direct mechanical stimulation of Merkel cells, which were subjected to 48-hour B1-receptor agonist treatment

**Figure 4.**
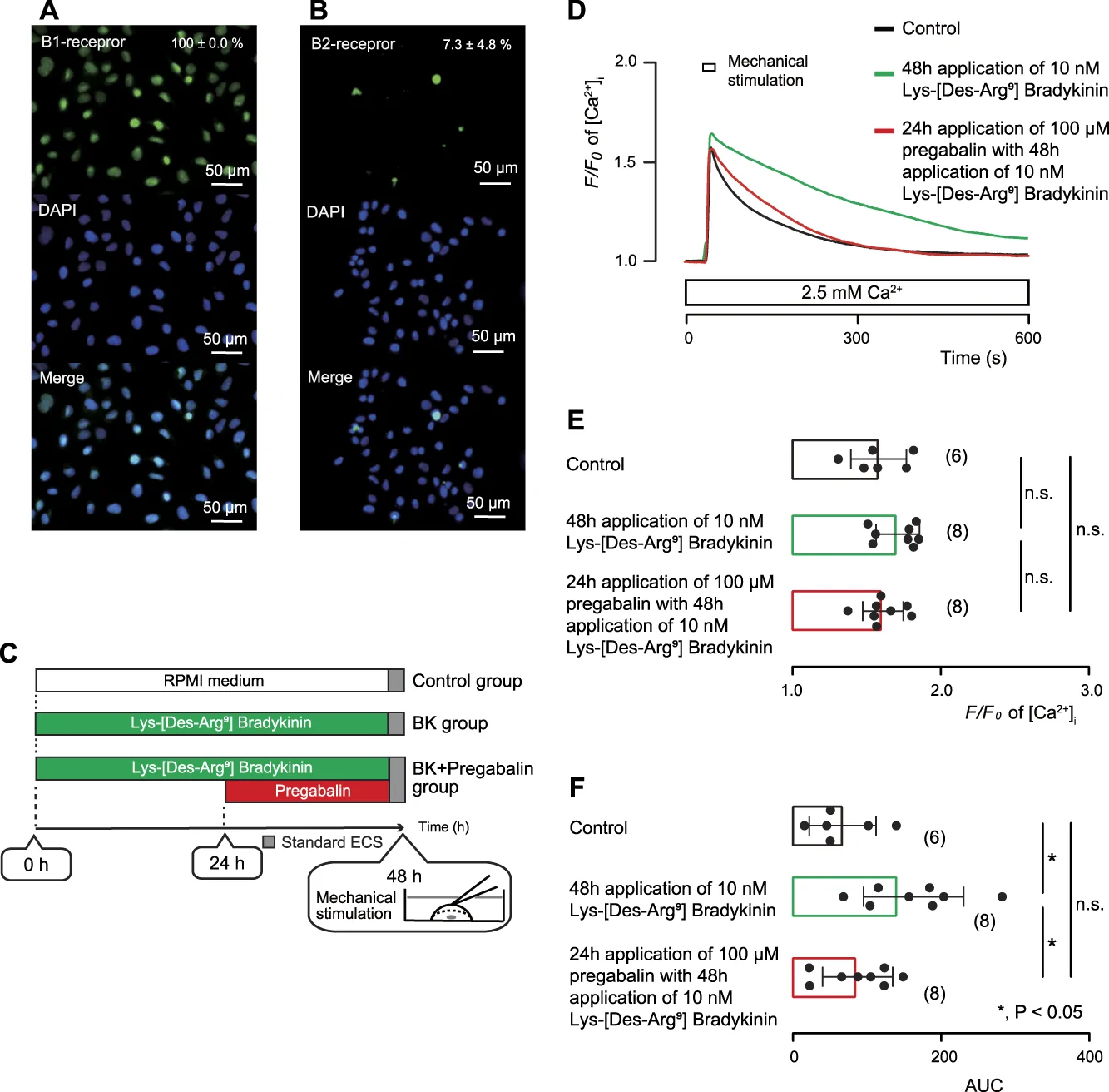
Effects of pregabalin on [Ca^2+^]_i_ increase induced by direct mechanical stimulation of human MCs pretreated with Lys-[Des-Arg^9^] bradykinin for 48 hours. (A and B) Human MCs exhibited positive immunoreactivity to the B1-receptor antibody (green; upper panel in A), but a weaker response to the B2-receptor antibody (green; upper panel in B). The expression rate of the B1-receptor was 100 ± 0.0% of MCs (N = 5), whereas the B2-receptor expression was observed at 7.3 ± 4.8% (N = 5). Nuclei are stained blue (DAPI, middle panels in A and B), and the lower panels in (A and B) display the merged images. The scale bars: 50 μm. No fluorescence was detected in the negative controls (not shown). (C) Protocols show the time period of application of Lys-[Des-Arg^9^] bradykinin (BK) and pregabalin. Each box represents the pharmacological reagents applied in the experiment (gray, standard ECS; green, RPMI medium with 48-hour application of 10-nM BK; red, RPMI medium with 24-hour application of 100-μM pregabalin, before mechanical stimulation). White boxes indicate culture condition with RPMI medium. Upper shows the control group; direct mechanical stimulations were applied to MCs that were cultured in RPMI medium for 48 hours. Middle represents the BK group; MCs were cultured for 48 hours in RPMI medium with 10-nM BK before direct mechanical stimulation. Lower indicates the BK + pregabalin group; MCs were cultivated in RPMI medium with 24-hour application of 10-nM BK and followed by 24-hour addition of 100-μM pregabalin with 10-nM BK before mechanical stimulation. (D) Traces obtained by averaging the independent traces (black line: N = 6, green line: N = 8, red line: N = 8) of transient increase in [Ca^2+^]_i_ during mechanical stimulations in each condition are shown. Direct mechanical stimulations were induced by vertical downward displacement of micropipette by 6.0 μm (white boxes at the top in D) in the control group (black line in D), the BK group (green line in D), and the BK + pregabalin group (red line in D). (E and F) Bar graphs show the peak *F/F*_*0*_ values (E) and AUC (F) after mechanical stimulation. Black boxes represent the control group (upper columns in E and F). Green boxes show values obtained in the BK group (middle columns in E and F). Red boxes represent values obtained in the BK + pregabalin group (lower columns in E and F). Each bar (in E and F) denotes the mean ± SD across the number of experiments shown in parenthesis, and each black circle represents each *F/F*_*0*_ value or AUC, respectively. Statistically significant differences between columns (solid lines) are indicated with asterisks: *P < 0.05; n.s., not significant. Statistical analysis was performed by the Kruskal–Wallis test followed by Dunn multiple comparison test in (E), and the ordinary 1-way ANOVA followed by Tukey multiple comparison test in (F). AUC, area under the curve; ECS, extracellular solution; MCs, Merkel cells.

We examined whether pregabalin affected [Ca^2+^]_i_ kinetics in MCs that were subjected to the long-lasting application of a B1-receptor agonist. We observed immunoreactivity of B1-receptor (100 ± 0.0%, N = 5) but rarely of B2-receptor (7.3 ± 4.8%, N = 5) in the MCs (Figs. 4A, B). To mimic tissue inflammatory and/or chronic pain conditions in vitro, we cultured MCs in a medium without (control group) or with 10-nM B1-receptor agonist Lys-[Des-Arg^9^] bradykinin (BK) for 48 hours (BK group) before direct mechanical stimulation. In addition, to examine the effects of pregabalin on MCs in the presence of BK (BK + pregabalin group; Fig. 4C), MCs were cultured for 24 hours in RPMI medium with 10-nM BK + 100-μM pregabalin after a 24-hour culture with 10-nM BK. In the control group, the peak value of mechanical stimulation–induced [Ca^2+^]_i_ increase was 1.58 ± 0.19, and the value of AUC was 67.2 ± 44.7 (N = 6, black line in Fig. 4D and black columns in Figs. 4E, F). In the BK group, the peak value of the increase in [Ca^2+^]_i_ by direct mechanical stimulation was 1.71 ± 0.15 in *F/F*_*0*_ units, and the value of AUC was 162.4 ± 67.1 (N = 8, green line in Fig. 4D and green columns in Figs. 4E, F). The value of AUC was significantly increased in the BK group compared to that in the control group (F_(2,19)_ = 0.77, *P* = 0.01; Figs. 4D, F), whereas there was no significant differences in the peak values of the increase in [Ca^2+^]_i_ between the BK and control groups (*P* = 0.43; Figs. 4D, E). In the BK + pregabalin group, the peak value of increase in [Ca^2+^]_i_ by direct mechanical stimulation was 1.61 ± 0.14 in *F/F*_*0*_ units, and the value of AUC was 87.1 ± 46.9 (N = 8, red line in Fig. 4D and red columns in Figs. 4E, F). The value of AUC, which was increased in the BK group (green column in Fig. 4F), was significantly decreased to the same level as that in the control group (black column) by the addition of pregabalin (BK + pregabalin group; F_(2,19)_ = 0.77, *P* = 0.03; red in Figs. 4D, F). In contrast, there was no significant difference in the peak value of [Ca^2+^]_i_ increase between the groups (*P* = 0.78; Figs. 4D, E). Statistical analysis was performed by the Kruskal–Wallis test followed by Dunn multiple comparison test for the peak values, and the ordinary 1-way ANOVA followed by Tukey multiple comparison test for the value of AUC.

### 3.5. Effect of pregabalin on [Ca^2+^]_i_ in Merkel cells subjected to 48-hour bradykinin application was suppressed by Na^+^–Ca^2+^ exchanger inhibitor

**Figure 5.**
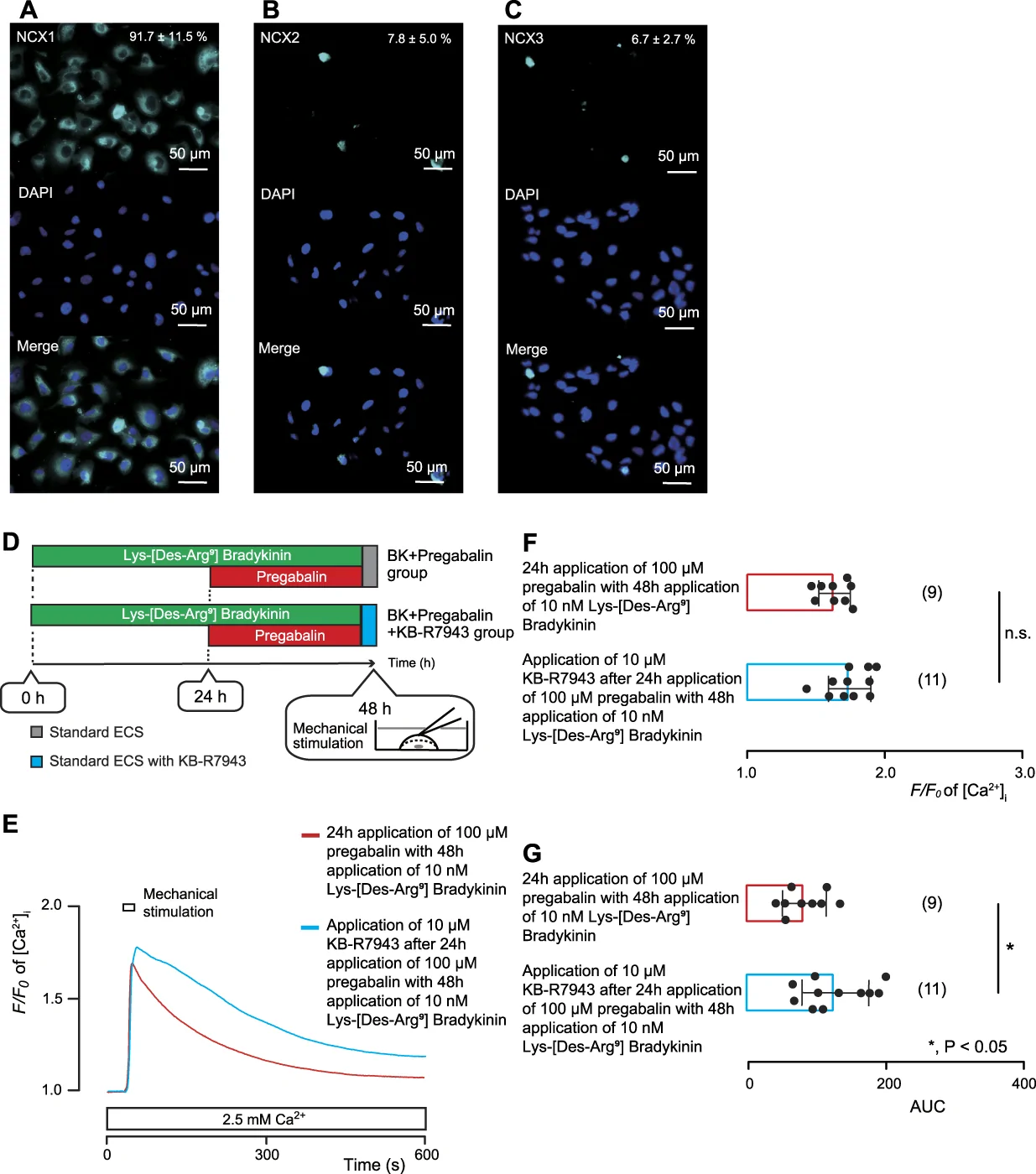
Effects of the Na^+^–Ca^2+^ exchanger inhibitor and pregabalin on direct mechanical stimulation–induced Ca^2+^ influx in human MCs. (A–C) Human MCs displayed strong immunoreactivity for the Na^+^–Ca^2+^ exchanger (NCX)-1 (light blue in the upper panel of A), whereas NCX2 and NCX3 showed minimal expression (light blue in the upper panels of B and C). The expression levels of the NCX isoforms in human MCs were as follows: NCX1, 91.7 ± 11.5% (N = 5); NCX2, 7.8 ± 5.0% (N = 5); and NCX3, 6.7 ± 2.7% (N = 5). Nuclei are indicated in blue (DAPI, middle panels of A-C), and the lower panels in (A-C) present merged images. Scale bar: 50 μm. No fluorescence was detected in the negative controls (not shown). (D) Protocols show the time period of application of Lys-[Des-Arg^9^] bradykinin (BK) and pregabalin. Each color box indicates the pharmacological reagents used in the experiment (gray, standard ECS; green, RPMI medium with 48-hour application of 10-nM BK; red, RPMI medium with 24-hour application of 100-μM pregabalin, before mechanical stimulation; blue, standard ECS with 10-μM KB-R7943). Upper shows the BK + pregabalin group; MCs were cultivated in RPMI medium with 24-hour application of 10-nM BK and followed by 24-hour addition of 100-μM pregabalin with 10-nM BK before mechanical stimulation. Lower represents the BK + pregabalin + KB-R7943 group; 10-μM KB-R7943 was applied just before mechanical stimulation to the MCs of the BK + pregabalin group. (E) Traces obtained by averaging the independent traces (red line: N = 9, blue line: N = 11) of transient increase in [Ca^2+^]_i_ during mechanical stimulations in each condition are shown. Direct mechanical stimulations were induced by vertical downward displacement of micropipette by 6.0 μm (white boxes at the top in E) in the BK + pregabalin group (N = 9, red line in E) or the BK + pregabalin + KB-R7943 group (N = 11, blue line in E). (F, G) Bar graphs show the peak values (F) and AUC (G) after mechanical stimulation (to 6.0 μm) in the BK + pregabalin group (red columns in F and G) or the BK + pregabalin + KB-R7943 group (blue columns in F and G). Each bar in F and G denotes the mean ± SD across the number of experiments shown in parenthesis, and each black circle represents each *F/F*_*0*_ value or AUC, respectively. Statistically significant differences between columns (solid lines) are indicated by asterisks: *P < 0.05; n.s., not significant; data were analyzed by the Welch *t* test. AUC, area under the curve; ECS, extracellular solution; MCs, Merkel cells.

To investigate the effect of pregabalin on Ca^2+^ extrusion pathway via NCX after mechanical stimulation–induced Ca^2+^ influx from the cells, we examined the immunoreactivity of NCX subtypes (NCX1, NCX2 and NCX3). Merkel cells showed immunopositive to NCX1 (91.7 ± 11.5%, N = 5) antibodies, whereas poorly immunopositive to NCX2 (7.8 ± 5.0%, N = 5) and NCX3 (6.7 ± 2.7%, N = 5) (Figs. 5A–C). Next, we applied KB-R7943, a NCX inhibitor, during direct mechanical stimulation to evaluate the functional role of NCX1 for MCs in pathological settings (Fig. 5D). When we applied 10-μM KB-R7943, the peak value of mechanical stimulation–induced [Ca^2+^]_i_ in MCs of the BK + pregabalin group was 1.75 ± 0.15 in *F/F*_*0*_ units, and the value of AUC was 130.7 ± 48.5 (N = 11, blue line in Fig. 5E and blue columns in Figs. 5F, G). Without KB-R7943, in the BK + pregabalin group, the peak value of mechanical stimulation–induced [Ca^2+^]_i_ in MCs was 1.64 ± 0.11 in *F/F*_*0*_ units, and the value of AUC after direct mechanical stimulation was 84.8 ± 31.9 (N = 9, red line in Fig. 5E and red columns in Figs. 5F, G). The value of AUC was significantly increased by the application of KB-R7943 compared to that in the BK + pregabalin group (*P* = 0.02). In contrast, there was no significant difference in the peak value of [Ca^2+^]_i_ increase by direct mechanical stimulation with or without KB-R7943 (*P* = 0.25). Data were analyzed by the Welch *t* test.

### 3.6. Effect of pregabalin on [Ca^2+^]_i_ in MCs subjected to 48-hour bradykinin application was suppressed by selective inhibitor of NCX1

**Figure 6.**
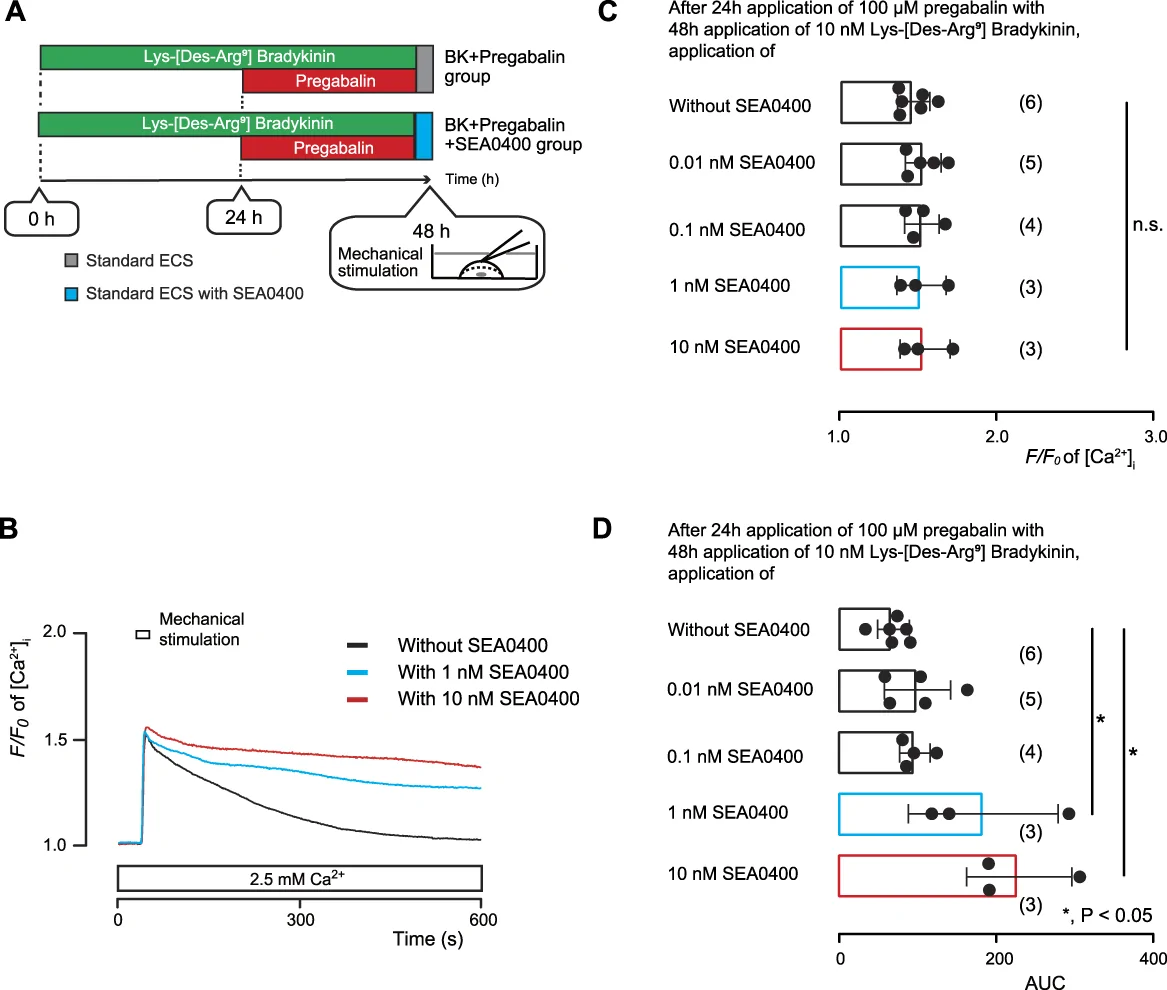
Effects of the selective inhibitor of NCX1 and pregabalin on direct mechanical stimulation–induced Ca^2+^ influx in human MCs. (A) Protocols show the time period of application of Lys-[Des-Arg^9^] bradykinin (BK) and pregabalin. Each color box indicates the pharmacological reagents used in the experiment (gray, standard ECS; green, RPMI medium with 48-hour application of 10-nM BK; red, RPMI medium with 24-hour application of 100-μM pregabalin, before mechanical stimulation; blue, standard ECS with SEA0400). Upper shows the BK + pregabalin group; MCs were cultivated in RPMI medium with 24-hour application of 10-nM BK and followed by 24-hour addition of 100-μM pregabalin with 10-nM BK before mechanical stimulation. Lower represents the BK + pregabalin + SEA0400 group; the different concentrations of SEA0400 were applied just before mechanical stimulation to the MCs of the BK + pregabalin group. (B) Traces obtained by averaging the independent traces (black line; without SEA0400, N = 6, blue line; with 1.0-nM SEA0400, N = 3, red line; with 10-nM SEA0400, N = 3) of transient increase in [Ca^2+^]_i_ during mechanical stimulation in each condition are shown. Direct mechanical stimulations were induced by vertical downward displacement of micropipette by 6.0 μm (white boxes at the top in B) in the BK + pregabalin group (N = 6, black line in B), with 1.0-nM SEA0400 group (N = 3, blue line in B) or with 10-nM SEA0400 group (N = 3, red line in B). (C and D) Bar graphs show the peak values (C) and AUC (D) after mechanical stimulation (to 6.0 μm) in the BK + pregabalin group (the first black columns in C and D), or the BK + pregabalin + SEA0400 group (0.01 nM; the second black columns, 0.1 nM; the third black columns, 1.0 nM; blue columns, 10 nM; red columns in C and D). Each bar in C and D denotes the mean ± SD across the number of experiments shown in parenthesis, and each black circle represents each *F/F*_*0*_ value or AUC, respectively. Statistically significant differences between columns (solid lines) are indicated by asterisks: *P < 0.05; n.s., not significant; data were analyzed by the Kruskal–Wallis test followed by Dunn multiple comparison test for AUC, and the ordinary 1-way ANOVA followed by Tukey multiple comparison test for the peak values. AUC, area under the curve; ECS, extracellular solution; MCs, Merkel cells; NCX, Na^+^–Ca^2+^ exchanger.

When we applied a series of concentrations of SEA0400, a selective inhibitor of NCX1, ranging from 0.01 nM to 10 nM, to MCs during direct mechanical stimulation (Fig. 6A), the peak value of mechanical stimulation–induced [Ca^2+^]_i_ in MCs (Fig. 6B) of the BK + pregabalin group were 1.54 ± 0.11 by 0.01nM (N = 5, the second black columns in Figs. 6C, D, *P* = 0.92), 1.53 ± 0.11 by 0.1 nM (N = 4, the third black columns in Figs. 6C, D, *P* = 0.96), 1.53 ± 0.16 by 1.0 nM (N = 3, blue line in Fig. 6B and blue columns in Figs. 6C, D, *P* = 0.97), and 1.55 ± 0.16 by 10 nM (N = 3, red line in Fig. 6B and red columns in Figs. 6C, D, *P* = 0.91) in *F/F*_*0*_ units, compared to the control value (1.48 ± 0.10 in *F/F*_*0*_ units [N = 6, black line in Fig. 6B and the first black columns in Figs. 6C, D]). There were no significant differences in the peak value of [Ca^2+^]_i_ increase with or without SEA0400 (F_(4,16)_ = 0.89). The values of AUC were increased to 98.88 ± 42.57 by 0.01 nM (*P* > 0.99), 95.45 ± 19.72 by 0.1 nM (*P* > 0.99), 183.40 ± 96.12 by 1.0 nM (*P* = 0.04), and 229.51 ± 67.53 by 10 nM (*P* = 0.007), compared to the control value (67.94 ± 20.45). The values of AUC were significantly increased by the application of 1.0-nM and 10-nM SEA0400 compared to the control. Data were analyzed by the ordinary one-way ANOVA followed by Tukey multiple comparison test for the peak values, and the Kruskal–Wallis test followed by Dunn multiple comparison test for the value of AUC.

### 3.7. Pregabalin did not suppress [Ca^2+^]_i_ increase by depolarizing stimulus

**Figure 7.**
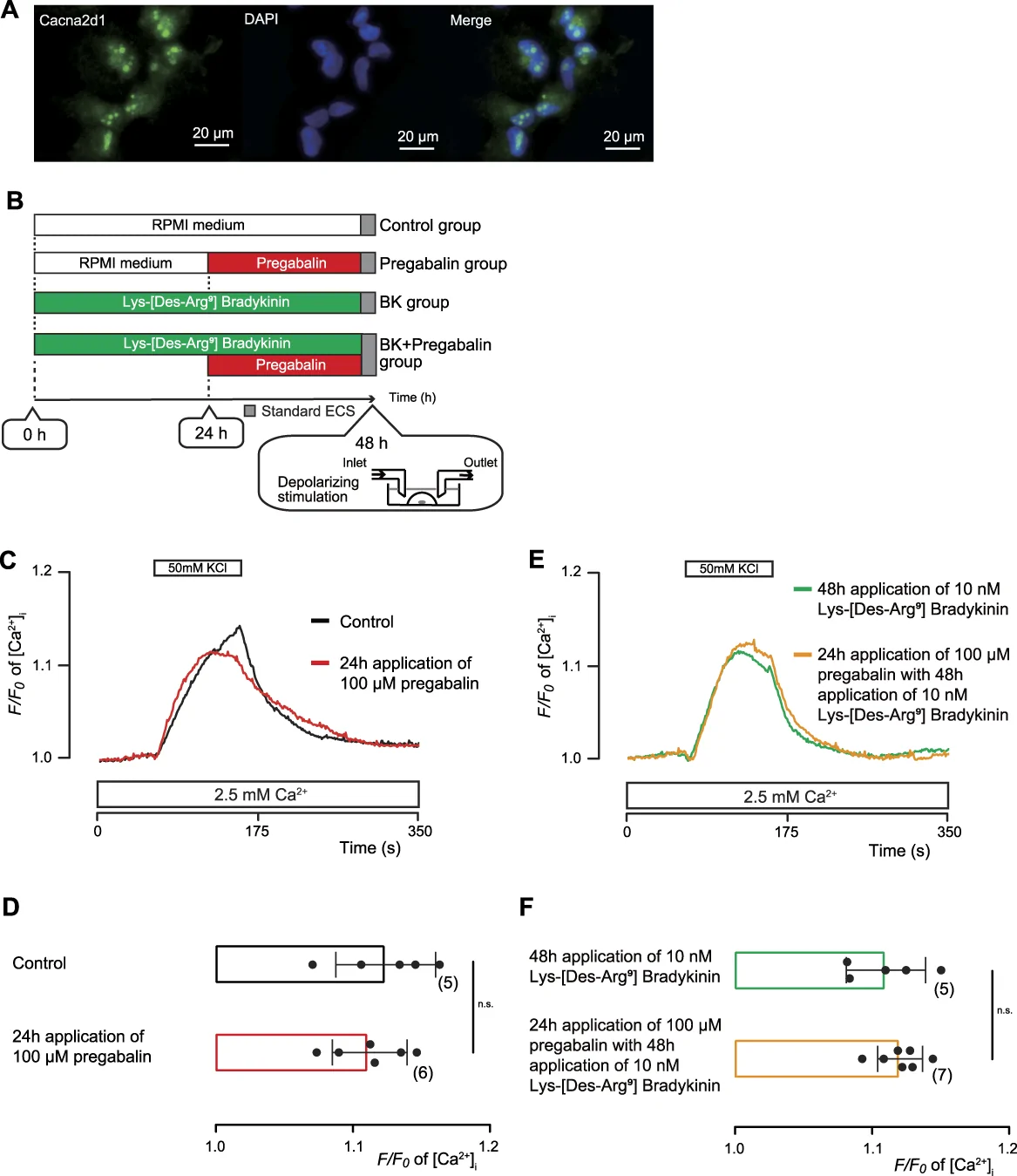
Effects of pregabalin on depolarizing stimulation-induced Ca^2+^ influx in human MCs. (A) Human MCs showed positive immunoreactivity for Cacna2d1, an alpha 2 delta protein of the auxiliary subunit of voltage-gated Ca^2+^ channels (green; leftmost panel in A). Nuclei are stained blue (DAPI, middle panel in A), and the rightmost panel displays the merged image. Scale bar: 20 μm. No fluorescence was detected in the negative controls (not shown). (B) Protocols show the time period of application of Lys-[Des-Arg^9^] bradykinin (BK) and pregabalin. Each color box indicates the pharmacological reagents in the experiment (gray, standard ECS; red, culture condition with RPMI medium including 100-μM pregabalin; green, RPMI medium with 48-hour application of 10-nM BK). White boxes indicate culture condition with RPMI medium. Upper represents the control group; depolarizing stimulations were provided to MCs that were cultured in RPMI medium for 48 hours. Second upper indicates the pregabalin group; MCs cultured in RPMI medium for 24 hours followed by 24-hour culture in RPMI containing 100-μM pregabalin, before depolarizing stimulation. Third upper shows the BK group; MCs cultured in RPMI medium with 10-nM BK for 48 hours before depolarizing stimulation. Lower indicates the BK + pregabalin group; MCs were cultured in RPMI medium with 24-hour application of 10-nM BK and further 24-hour addition of 10-nM BK with 100-μM pregabalin to the medium before depolarizing stimulation. MCs cultured under each protocol condition were incubated in the standard ECS containing 10 mmol/L fura-2 acetoxymethyl ester and 0.1% (wt/vol) pluronic acid F-127 at 37°C for 60 minutes, then rinsed with fresh standard ECS. The high-K^+^ solution containing (in mM) (91 NaCl, 50 KCl, 2.5 CaCl_2_, 0.5 MgCl_2_, 10 HEPES, 10 glucose, and 12 NaHCO_3_) was used for depolarizing stimulus. Standard ECS and high-K^+^ solution were applied from the inlet by a rapid gravity-fed perfusion system with flow rate of 0.5 to 1.0 mL/minute and aspirated using a vacuum pump. (C and E) Representative traces of transient increase in [Ca^2+^]_i_ in response to 50 mM high-K^+^ solution in presence of extracellular Ca^2+^ (2.5 mM) (white boxes at bottom), in the control group (black line in C), the pregabalin group (red line in C), the BK group (green line in E), or the BK + pregabalin group (orange line in E). White boxes at the top indicate periods of high-K^+^ solution addition to the external solution. (D and F) Bar graph shows the high-K^+^ solution–induced increase in [Ca^2+^]_i_, in the control group (black column in D), the pregabalin group (red column in D), the BK group (green column in F), or the BK + pregabalin group (orange column in F). Each bar (in D and F) denotes the mean ± SE across the number of experiments shown in parenthesis, and each black circle represents each *F/F*_*0*_ value. No statistically significant differences between columns (solid lines) are indicated as n.s.; data were analyzed by the Welch *t* test. ECS, extracellular solution; MCs, Merkel cells.

We also observed the immunoreactivity of Cacna2d1 in MCs (green in Fig. 7A), a subunit of the voltage-gated Ca^2+^ channel, which is known as the binding site of pregabalin. To examine the effects of pregabalin on voltage-gated Ca^2+^ channels, we performed depolarizing stimulation using a high-K^+^ solution under various conditions (Fig. 7B). We observed transient [Ca^2+^]_i_ increases to the peak value of 1.12 ± 0.02 in *F/F*_*0*_ units in the control group (N = 5, black line in Fig. 7C and black column in Fig. 7D). The peak value of [Ca^2+^]_i_ increase due to depolarizing stimulation after the application of 100-μM pregabalin for 24 hours (pregabalin group) was 1.11 ± 0.01 in *F/F*_*0*_ units (pregabalin group in Fig. 7B; N = 6, red line in Fig. 7C and red column in Fig. 7D), showing that [Ca^2+^]_i_ by depolarizing stimulation was not suppressed by long-term application of pregabalin (*P* = 0.57).

In the cells that were cultured with BK for 48 hours (BK group in Fig. 7B), the peak value of [Ca^2+^]_i_ increase due to depolarizing stimulation was 1.10 ± 0.01 in *F/F*_*0*_ units (N = 5, green line in Fig. 7E and green column in Fig. 7F). In the cells cultured with BK and pregabalin (BK + pregabalin group in Fig. 7B), the peak value of [Ca^2+^]_i_ increase due to depolarizing stimulation was 1.12 ± 0.01 in *F/F*_*0*_ units (N = 7, orange line in Fig. 7E and orange column in Fig. 7F). The peak value of [Ca^2+^]_i_ increase due to depolarizing stimulation was not suppressed by the application of pregabalin, even 48 hours after the application of BK to MCs (*P* = 0.49). Data were analyzed by the Welch *t* test.

## 4. Discussion

The current study revealed the site of action of pregabalin in MCs. The Piezo2 channel is prominently expressed in sensory neurons and sensory receptors, such as the dorsal root ganglia, and trigeminal ganglia, as well as MCs.^1^ The Piezo2 is a channel also important for mechanotransduction in MCs,^39^ which is required for touch sensation.^8,11^ Furthermore, the Piezo2 channel was reported to be involved in the development of allodynia in a Piezo2 knockdown model.^11^ In the present study, we observed Piezo2 immunoreactivity, as an MC marker protein. Therefore, it is suggested that the [Ca^2+^]_i_ induced by direct mechanical stimulation of MCs was due to Piezo2 activation.

Mechanical stimulation–induced [Ca^2+^]_i_ response in MCs was not inhibited by pregabalin under physiological conditions, indicating that pregabalin did not have any effects on the mechanosensitive processes. We also hypothesized that inflammatory and/or chronic pain conditions are necessary to examine the modulatory effect of pregabalin on the mechanotransduction mechanism in MCs. The activation of BK receptors is known to promote neuropathic pain.^23^ Intrathecal administration of BK induces mechanical allodynia.^35^ A decrease in pain threshold was observed after intraplantar injection of BK.^13^ Therefore, we attempted to produce an allodynia-like model by applying BK to MCs in vitro. In this study, we observed the immunoreactivity of B1- but not B2-receptor antibodies in MCs; therefore, we investigated Lys-[Des-Arg^9^] BK, a selective agonist of the B1-receptor. B1-receptors are involved in the chronic phase of pain response, whereas B2-receptors are involved in the acute phase of pain response.^9,10^ After inflammation or nerve injury, B1-receptor expression increased in not only sensory neurons^25^ but also peripheral skin.^38^ Spinal nerve–injured rats showed strong nociceptive responses to intraplantar injections of selective B1 receptor agonists, whereas sham-operated rats had no response to B1 receptor agonists.^38^ In the BK group in the present study, the value of AUC was increased. Since the value of AUC indicates the extrusion time of intracellular Ca^2+^ after the increase in [Ca^2+^]_i_, the result suggested that BK induces [Ca^2+^]_i_ accumulation via the moderation of Ca^2+^ extrusion proteins. Extrusion of intracellular Ca^2+^ after the increase in [Ca^2+^]_i_ is delayed by inflammation^27^ and diabetic neuropathy^16,26^; previous reports were consistent with our current results. In this study, B1 receptors were highly expressed in MCs, and reproduction of the allodynia-like model by applying a selective B1 agonist seemed quite possible.

Intracellular Ca^2+^ levels are precisely regulated by Ca^2+^ mobilization and extrusion. Ca^2+^ extrusion out of the cell membranes involves either high-affinity low-capacity plasma membrane Ca^2+^-ATPase (PMCA) or low-affinity high-capacity NCX.^5^ Plasma membrane Ca^2+^-ATPase is responsible for extruding Ca^2+^ at rest,^6^ and NCX is responsible for extruding Ca^2+^ after large rise in [Ca^2+^]_i_.^37^ In the BK + pregabalin group, the value of AUC was decreased to the same level as in the control group. When we applied KB-R7943 to the BK + pregabalin group, the effect of pregabalin was abolished. The results suggested that pregabalin might functionally and acutely normalize the decreased activity in NCX caused by BK. The activity of NCX in nociceptive neurons decreases during inflammatory response.^32^ Furthermore, the time course of the decrease in NCX activity and the inflammation-induced decrease in the nociceptive threshold were consistent.^32^ These findings supported our results showing that the pregabalin likely modulates/normalizes and/or accelerates impaired NCX activity by BK-induced cellular dysfunction. Based on these results, it is unlikely that BK modulates NCX, particularly NCX1. It was reported that B1 receptor activation activates intracellular Ca^2+^ influx via reverse mode (Ca^2+^ influx mode) of NCX.^17^ Therefore, we cannot exclude the possibility that pregabalin suppressed NCX reverse activity in this study, but in that case, the value of AUC would not be increased by the administration of KB-R7943 and SEA0400. Three subtypes of NCX are known,^31^ and immunofluorescence staining showed predominant NCX1-immunopositivity in MCs, whereas MCs were also immunopositive for Cacna2d1; however, Cacna2d1 expression was mainly observed in the nucleolus and rarely in the cell membrane or cytoplasm. Cacna2d1 is known to be the site of action of pregabalin at the central presynaptic terminals. Furthermore, pregabalin did not suppress [Ca^2+^]_i_ increase upon depolarizing stimulation using a high-K^+^ solution. Therefore, pregabalin might act on NCX1 rather than Cacna2d1 in MCs.

In the present study, however, we did not investigate the effect of pregabalin on the neurons. When we subjected human MCs to long-term BK treatment, the decay phase of [Ca^2+^]_i_ was prolonged, suggesting the intracellular accumulation of [Ca^2+^]_i_ in MCs. This could decrease the threshold of mechanosensitivity in human MCs to induce abnormal or augmented neurotransmitter release from MCs. It has been shown that glutamate, a neurotransmitter released from MCs, activates the N-methyl-d-aspartate receptors on Aβ afferent as MC-neurite complexes to mediate sensory transmission.^15^ The acquired low-threshold properties of MCs could be involved in the allodynia, in which the touch or pain sensation occurs even if the stimuli are quite low. However, in this study, we could not investigate synaptic (intercellular) communication from MCs to afferent neurons, and further study will be required in the future using an MCs-neurons coculture system.

Our previous study reported that the application of Lys-[Des-Arg^9^] BK evoked transient increases in [Ca^2+^]_i_ in rat trigeminal ganglion neurons via activation of the intracellular Ca^2+^ releasing pathway that is mediated by ryanodine receptors in either the presence or absence of extracellular Ca^2+^.^34^ We did not examine in detailed effect of BK to intercellular Ca^2+^ mobilization pathway in the MCs, further study will be needed to clarify this.

In addition, previous study has reported that administration of pregabalin at concentrations of 10 μM to 100 μM suppressed nerve stimulation–induced soleus muscle contraction, with maximum effect at 100μM in vitro.^20^ Therefore, in this study, we used 100 μM of applied concentration of pregabalin, but the final concentration of pregabalin in the basal cell layer when the pregabalin was topically application to the epithelial tissue is not clear.

In conclusion, pregabalin may acutely modulate [Ca^2+^]_i_ homeostasis by increasing Ca^2+^ extrusion activity via the plasma membrane NCX1 to rescue the cells from intracellular Ca^2+^ overload caused by BK-induced Ca^2+^ accumulation. This could also maintain the mechanosensitivity threshold in human MCs under normal or physiological conditions. We, therefore, supported the previous report of topical administration of pregabalin to epithelial tissue with neuropathic pain exerting an analgesic effect. Topical administration of pregabalin to the peripheral epithelial tissue in patients with neuropathic pain may reduce persistent pain and side effects.

## Disclosures

The authors have no conflict of interest to declare.

## Supplemental digital content

Supplemental digital content associated with this article can be found online at http://links.lww.com/PR9/A366.

## Supplementary Material
