# Supplementary material for: Pregabalin acts on Na+–Ca2+ exchanger, which promotes Ca2+ extrusion from human Merkel cell line
Source: Pain Rep. 2025 Dec 18;11(1):e1381. doi: 10.1097/PR9.0000000000001381 (PMC12721786; doi:10.1097/PR9.0000000000001381)
Supplement: Supplementary file 1 [file painreports-11-e1381-s001.pdf]

Supplementary Methods

1. Solutions and reagents

A solution containing 136 mM NaCl, 5 mM KCl, 2.5 mM $CaCl_2$, 0.5 mM $MgCl_2$, 10 mM 2-[4-(2-hydroxyethyl)-1-piperazinyl] ethanesulfonic acid (HEPES), 10 mM glucose, and 12 mM $NaHCO_3$ (pH7.4 with Tris[hydroxymethyl] aminomethane) was used as the standard extracellular solution (ECS). Depolarizing stimulus was induced by the application of a high-K^+ solution (91 mM NaCl, 50 mM KCl, 2.5 mM $CaCl_2$, 0.5 mM $MgCl_2$, 10 mM HEPES, 10 mM glucose, and 12 mM $NaHCO_3$; pH7.4 with Tris[hydroxymethyl]aminomethane).
Pregabalin was obtained from Sigma-Aldrich (St. Louis, MO, USA). The bradykinin receptor B1 (B1-receptor) agonist Lys-[Des-Arg^9] bradykinin (BK) and the Na^+-Ca^{2+} exchanger (NCX) inhibitor KB-R7943 were obtained from Tocris Bioscience (Bio-Techne, Minneapolis, MN, USA). The concentration of BK [1] and KB-R7943 [2] was determined according to the described previous studies. The NCX1 inhibiter SEA0400 was obtained from Selleck Biotechnology (Kanagawa, Japan). The concentration of SEA0400 was determined as described previously. [3] Stock solutions were prepared by dissolving the reagents in dimethyl sulfoxide (DMSO) for Lys-[Des-Arg^9] bradykinin and SEA0400 and in MilliQ water for KB-R7943 and pregabalin. For depolarizing stimulation, high-K^+ solution was applied to cells using a rapid gravity-fed perfusion system with flow rate of 0.5-1.0 ml/min (VC-8 PTFE; Warner Instruments, Holliston, MA, USA). Solution changes were completed within ~20 milliseconds.

2. Immunofluorescence

The Merkel cells (MCs) were cultured in 8-well glass chambers (AGC TECHNO GLASS, Shizuoka, Japan) and maintained at 37 °C and 5% CO_2. The cells were fixed with 4% paraformaldehyde (FUJIFILM Wako Pure Chemical, Osaka, Japan) and washed with phosphate-buffered saline (PBS; Thermo Fisher Scientific, Waltham, MA, USA). After 60 min

of incubation with 0.1–0.3% Triton X-100 (Sigma Aldrich) and a blocking reagent (Nacalai Tesque, Kyoto, Japan) at room temperature (28 °C), the following primary antibodies were added and incubated at 4 °C overnight: mouse monoclonal anti-cytokeratin 8 (sc-8020), mouse monoclonal anti-cytokeratin 20 (sc-271183), and mouse monoclonal anti- voltage-gated Ca^{2+} channel auxiliary subunit alpha2delta-1 (sc-271697) (Santa Cruz Biotechnology, Dallas, TX, USA; 1:100 for each), mouse monoclonal anti-cytokeratin 14 (Abcam, Cambridge, United Kingdom; ab-77684, 1:100), rabbit polyclonal anti-Piezo2 (Santa Cruz Biotechnology; sc-84818, 1:100), rabbit polyclonal anti-B1 (#ABR-001) and B2 receptor (#ABR-012) (Alomone Labs, Jerusalem, Israel; 1:100 for each). We also added and incubated with antibodies of goat polyclonal anti-NCX1 (sc-30306), -NCX2 (sc-33528), and -NCX3 (sc-48896) (Santa Cruz Biotechnology; 1:100 for each). The cells were then incubated with Alexa Fluor® 488 or Alexa Fluor® 568 conjugated secondary antibodies (all from Thermo Fisher Scientific) for 60 min at room temperature (28 °C). MCs were mounted in a mounting medium containing 4,6-diamidino-2-phenylindole (DAPI; Abcam; Ab104139). DAPI was used to stain the nuclei. We observed the immunofluorescence images using a fluorescence microscope (BZ-X710; Keyence, Osaka, Japan).

3. Measurement of intracellular free Ca^{2+} concentration

For the measurements of intracellular free Ca^{2+} concentration ($[Ca^{2+}]_i$) induced by both direct mechanical and depolarizing stimulation, we loaded fura-2 acetoxymethyl ester (10 μM; Dojindo, Kumamoto, Japan) with pluronic acid F-127 (0.1% (w/v); Thermo Fisher Scientific) into MCs cultured with a 35 mm cell culture imaging dish (μ-Dish; ibidi GmbH, Gräfelfing, Germany) for 60 min at 37 °C. Next, we rinsed the MCs in a dish with fresh ECS and placed the culture dish on a microscope stage (Axio Observer 7; Carl Zeiss, Oberkochen, Germany). We measured fura-2 fluorescence at 510 nm using alternating excitation wavelengths of 340

(F340) and 380 nm (F380) (HCImage Imaging software; Hamamatsu Photonics, Shizuoka, Japan). The $[Ca^{2+}]_i$ was determined using the fluorescence ratio ($R_{F340/F380}$) of F340 to F380, which was represented in F/F_0 units, where the $R_{F340/F380}$ value (F) was normalized to the resting value (F_0). The F/F_0 baseline was arbitrarily set at 1.0. The software controlled an intensified charge-coupled device camera system (Hamamatsu Photonics) and a selector for the excitation wavelength. We performed all the experiments at room temperature (28 °C).

References

[1]. Kawaguchi A, Sato M, Kimura M, Yamazaki T, Yamamoto H, Tazaki M, Ichinohe T, Shibukawa Y. Functional expression of bradykinin B1 and B2 receptors in neonatal rat trigeminal ganglion neurons. Front Cell Neurosci. 2015;9:229. doi: 10.3389/fncel.2015.00229.

[2]. Kuroda H, Sobhan U, Sato M, Tsumura M, Ichinohe T, Tazaki M, Shibukawa Y. Sodium-calcium exchangers in rat trigeminal ganglion neurons. Mol Pain. 2013;9:22. doi: 10.1186/1744-8069-9-22.

[3]. Tsumura M, Okumura R, Tatsuyama S, Ichikawa H, Muramatsu T, Matsuda T, Baba A, Suzuki K, Kajiya H, Sahara Y, Tokuda M, Momose Y, Tazaki M, Shimono M, Shibukawa Y. Ca^{2+} extrusion via Na^{+}-Ca^{2+} exchangers in rat odontoblasts. J Endod. 2010;36:668-74. doi: 10.1016/j.joen.2010.01.006.
